# Origin of B chromosomes in *Characidium alipioi* (Characiformes, Crenuchidae) and its relationship with supernumerary chromosomes in other *Characidium* species

**DOI:** 10.3897/CompCytogen.v11i1.10886

**Published:** 2017-01-20

**Authors:** Érica Alves Serrano, Ricardo Utsunomia, Patrícia Sobrinho Scudeller, Claudio Oliveira, Fausto Foresti

**Affiliations:** 1 Departamento de Morfologia, Instituto de Biociências, Universidade Estadual Paulista, Distrito de Rubião Junior, s/n, 18618-970, Botucatu, São Paulo, Brazil

**Keywords:** Microdissection, Chromosome painting, FISH, B chromosomes

## Abstract

B chromosomes are apparently dispensable components found in the genomes of many species that are mainly composed of repetitive DNA sequences. Among the numerous questions concerning B chromosomes, the origin of these elements has been widely studied. To date, supernumerary chromosomes have been identified in approximately 60 species of fish, including species of the genus *Characidium* Reinhardt, 1867 in which these elements appear to have independently originated. In this study, we used molecular cytogenetic techniques to investigate the origin of B chromosomes in a population of *Characidium alipioi* Travassos, 1955 and determine their relationship with the extra chromosomes of other species of the genus. The results showed that the B chromosomes of *Characidium alipioi* had an intraspecific origin, apparently originated independently in relation to the B chromosomes of *Characidium gomesi* Travassos, 1956 *Characidium pterostictum* Gomes, 1947 and *Characidium oiticicai* Travassos, 1967, since they do not share specific DNA sequences, as well as their possible ancestral chromosomes and belong to different phylogenetic clades. The shared sequences between the supernumerary chromosomes and the autosommal sm pair indicate the origin of these chromosomes.

## Introduction

B or supernumerary chromosomes are extra genomic elements in addition to the standard chromosomal set (A) and are found in approximately 15% of eukaryotic organisms (Camacho 2005). In general, B chromosomes are derived from the A chromosomes of their own species (intraspecific origin) or closely related species (interspecific origin) (Banaei-Moghaddam et al. 2015). The intraspecific origin of B chromosomes was demonstrated in maize (Lamb et al. 2005; Peng et al. 2011), locusts (Teruel et al. 2010), rye (Martis et al. 2012) and fishes (Mestriner et al. 2000; Silva et al. 2013; Valente et al. 2014; Utsunomia et al. 2016). On the other hand, there are also cases in which B chromosomes may have arisen spontaneously in response to new genomic conditions such as interspecific hybridization, which has been observed in grasses of the genus *Coix* Linné, 1753 (Sapre and Deshepande 1987), the fish *Poecilia formosa* Girard, 1859 (Schartl et al. 1995) and the wasp *Nasonia vitripennis* Walker, 1836 (McAllister and Werren 1997).

A significant evolutionary feature of B chromosomes is the accumulation of repetitive DNA sequences (Camacho 2005; Houben et al. 2014; Banaei-Moghaddam et al. 2015). The cytogenetic and/or cytogenomic data about this type of DNA have been very informative for understanding the origin and evolution of B chromosomes. The findings using fluorescence *in situ* hybridization (FISH) techniques and genomic sequences analysis showed the intraspecific origin of B chromosomes and the presence and expression of intact genes in these elements in fishes (Silva et al. 2013, 2016; Utsunomia et al. 2016; Valente et al. 2014), as also observed in rye (Banei-Moghaddam et al. 2015).

Among genomes of fish species bearing B chromosomes, the genus *Characidium* Reinhardt, 1867 exhibits interesting cytogenetic features; the B chromosomes probably originated independently in the different species of this group, whereas the heteromorphic ZZ/ZW sex chromosomes seem to have originated once in the genus. Therefore, the B chromosomes of *Characidium oiticicai* Travassos, 1967 originated interspecifically, whereas in *Characidium gomesi* Travassos, 1956 and *Characidium pterostictum* Gomes, 1947, these elements have an intraspecific origin from the sex chromosomes (Pansonato-Alves et al. 2014, Serrano et al. 2016). In the present study, a novel occurrence of B chromosomes is described for *Characidium alipioi*, a species in which these elements were not found until now. In addition, the origin and evolution of these elements were studied using conventional cytogenetic techniques, including C-banding, microdissection, chromosome painting and fluorescence *in situ* hybridization with repetitive DNA probes.

## Material and methods

### Origin of the fishes/individuals, karyotype analysis and DNA extraction

A total of 19 *Characidium alipioi* samples were analyzed (9 females and 10 males) from the Ribeirao Grande river, Paraíba do Sul River Basin, Pindamonhangaba, São Paulo (22°49'00.3"S 45°25'23.7") (Table 1). The animals were collected in accordance with Brazilian environmental laws for the permission to collect issued by MMA/IBAMA/SISBIO, number 3245. The collection procedures, maintenance and analysis of the animals were performed in accordance with the international regulations for animal experiments, followed by the Universidade Estadual Paulista (CEEAA/IBB/UNESP protocol number 304). The samples were identified and deposited into the fish collection of the Biology and Genetics Laboratory of Fish at Botucatu, São Paulo, Brazil, under number 22287.

**Table 1.** B chromosome polymorphisms in *Characidium alipioi*. Samples: number of males (M) and females (F) analyzed. Prevalence: the total and sex-specific percentage of individuals carrying B chromosomes.

Samples	Samples number with up to:					Prevalence
	**50**	**51**	**52**	**53**	**54**	
9 F	5	2	0	1	1	44%
10 M	5	3	1	0	1	50%
**19 Total**	**10**	**5**	**1**	**1**	**2**	**45%**

To perform the cytogenetic preparations, the animals were anesthetized and dissected, and mitotic chromosome preparations were obtained following the protocol of Foresti et al. (1981). C-banding was performed according to the protocol described by Sumner (1972). Chromosome morphology was determined according to Levan et al. (1964), and the chromosomes were classified as metacentric (m), submetacentrics (sm) and subtelocentric (st) and organized in the karyotype by descending size.

DNA extraction was performed using the Wizard Genomic DNA Purification Kit (Promega) according to the manufacturer’s instructions.

### Mitochondrial DNA analysis

Amplification and partial sequencing of cytochrome oxidase c subunit 1 (COI) and cytochrome b (Cyt B) was performed to identify the specimens. The primers used were as follows: Cyt BL 14841 (5’-CCA TCC CAA ATC ACT GCA TGA TGA AA-3 ‘) and Cyt BH 15915b (5’-AAC CTC TCT CGA GCT GAT TACAAG AC -3’) (Kocher et al. 1989) for Cyt B and COI L6252-Asn (5’-AAG GCG GGG AAA GCC GCC GCA G -3 ‘) and H7271-COXI (5’-TCC TAT GCC GAA GTA TGG TTC TTT T 3’) for COI (Melo et al. 2011). The sequences were analyzed using Geneious Pro v8.05 software, and the alignment was performed with the algorithm MUSCLE (Edgar 2004). The average distances between the sequences were calculated using the “pairwise deletion” option in MEGA 4.0 software (Tamura et al. 2007).

### Microdissection and preparation of chromosomal probe

Chromosome microdissection was performed in an Eppendorf TransferMan NK2 micromanipulator coupled with a Zeiss Axiovert 100 microscope. For chromosome painting, ten B chromosomes were microdissected from the cytogenetic preparations of the samples from each species (*Characidium alipioi*, *Characidium gomesi* and *Characidium oiticicai)* carrying one extra chromosome. The probes for *Characidium alipioi*, *Characidium gomesi* and *Characidium oiticicai* denoted CaB, CgB and CoB, respectively.

Microdissected DNA from each species was placed into a tube containing 9 µL of DNase-free ultrapure water and amplified using the GenomePlex Single Cell Whole Genome Amplification Kit (wga4 Sigma) (Gribble et al. 2004). After the initial amplification, the DNA probes CaB, CgB and CoB were generated and were then labeled with Digoxigenin-11-dUTP (Roche Applied Science) using the GenomePlex Whole Genome Amplification Reamplification Kit (wga3 Sigma) according to the manufacturer’s protocol.

### Repetitive DNA probe

Probes for 18S and 5S rDNA, U2 snDNA and histone H3 genes were obtained using PCR from the *Characidium alipioi* genome with previously described primers. Sequences for 5S and 18S rDNA were amplified using the *primers* 5SA (5’-TCA ACC AAC CAC AAA GAC ATT GGC AC-3’) and 5SB (5’-TAG ACT TCT GGG TGG CCA AAG GAA TCA-3’) (Pendás et al. 1995), 18S6F (5’-CTC TTT CGA GGC CCT GTA AT-3’) and 18S6R (5’-CAG CTT TGC AAC CAT ACT CC-3’) (Utsunomia et al. 2016). To amplify the H3 histone gene were utilized the following *primers*, H3F (5’-ATG GCT CGT ACC AAG CAG ACV GC-3’) and H3R (5’-ATA TCC TTR GGC ATR ATR GTG AC-3’) (Colgan et al. 1998). The U2 snDNA was amplified by the *primers* U2F (5’-ATC GCT TCT CGG CCT TAT G-3’) and U2R (5’-TCC CGG CGG TAC AAT TGC A-3’) (Silva et al. 2015).. The 18S rDNA, U2 snDNA and histone H3 probes were labeled with Digoxigenin-11-dUTP (Roche), and the 5S rDNA probe was labeled with biotin-16-dUTP (Roche). Oligonucleotide probes sequences containing microsatellite (CA)_15_, (GA)_15_ and (GAG)_10_ were labeled directly with TAMRA during the synthesis process by Sigma according to methods described by Kubat et al. (2008).

### Fluorescent in situ hybridization (FISH)

For the FISH experiments, the prehybridization conditions were performed according to procedures described by Pinkel et al. (1986). Posthybridization washes were performed according to the applied probe: (i) the slides probed with rDNAs, snDNA, histone and B chromosome probes were washed in 0.2× SSC/15% formamide for 20 min at 42°C, followed by a second wash in 0.1× SSC for 15 min at 60°C and a final wash at room temperature in 4× SSC/0.5% Tween for 10 min; probe detection was performed using avidin-FITC and anti-digoxigenin-rhodamine; and (ii) the slides probed with oligonucleotides were washed in 2× SSC for 5 min, followed by a second wash in 1× PBS for 1 min. Chromosomes were counterstained with DAPI (Vector Laboratories, Burlingame, Calif., USA). The images were digitally captured using Image Pro Plus 6.0 software (Media Cybernetics) with the appropriate filters on an epifluorescence microscope (Olympus BX61) equipped with an Olympus DP70 camera. The final composition of the images was performed using Adobe Photoshop CS6 image editor software with the image and uniform size scales.

## Results

### Analysis of mitochondrial DNA

**Figure 1.**
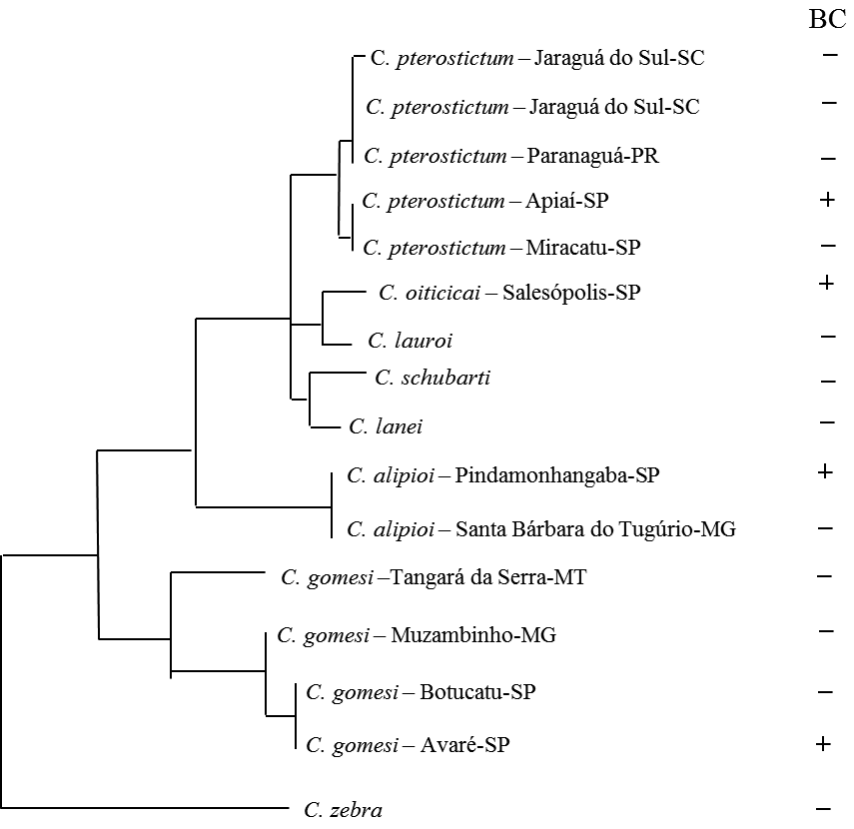
*Characidium* phylogeny adapted from Pansonato-Alves et al. (2014) and positioned for the population analyzed here. BC: presence or absence of B chromosomes.

Analysis of mitochondrial DNA was performed in order to make a correct identification of the specimens, besides that, the position of the clades could be informative to discuss aspects of the origin of the B chromosomes in the genus. The average distance analysis of the COI and CytB sequences obtained in this study and other species taken from GenBank showed high similarity between the *Characidium alipioi* sequences of Santa Bárbara do Tugúrio-MG and the specimens analyzed here (Suppl. materials 1, 2), which confirmed the taxonomic status of the analyzed samples (Figure 1).

### Chromosomal analysis

**Figure 2.**
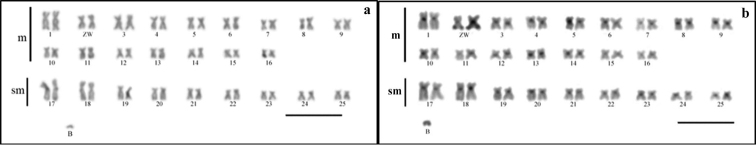
*Characidium alipioi* karyotypes arranged from mitotic metaphases after to conventional Giemsa-staining (**a**) and C-banding (**b**). Bar = 10 µm.

The analyzed *Characidium alipioi* individuals showed diploid chromosome number 2n = 50 and karyotype composed of 32 m + 18 sm with heteromorphic ZZ/ZW sex chromosomes, which was similar to the findings in other species of the genus (Figure 2). In addition, cells bearing 0-4 B chromosomes were observed in 45% of the individuals (Table 1). The B chromosomes were mitotically unstable, once the number of these chromosomes can variete between cells of the same individual. C-banding showed constitutive heterochromatin blocks in the pericentromeric region of all chromosomes and a conspicuous distal block in the longer arm of the Z chromosome. The W and B chromosomes were entirely heterochromatic (Figure 2b).

### Chromosome painting

**Figure 3.**
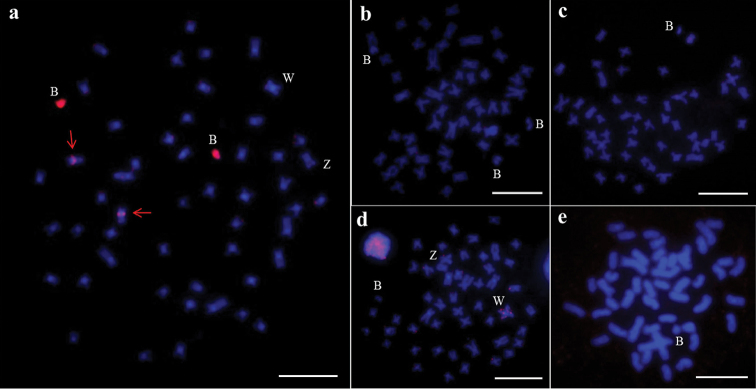
Cross-species chromosome painting. CaB probe in *Characidium alipioi* (**a**), *Characidium gomesi* (**b**) and *Characidium oiticicai* (**c**) and with the CgB (**d**) and CoB (**e**) probes in *Characidium alipioi*.

Chromosome painting performed in the *Characidium alipioi* chromosomes with the CaB probe showed signals on the entire length of the B chromosomes and the pericentromeric region of the sm chromosome pair nº 19 of the A complement (Figure 3a). On the other hand, this same probe did not reveal any signals on the *Characidium gomesi* and *Characidium oiticicai* chromosomes (Figure 3b and c). Similar findings were observed by hybridization with the CoB probe in *Characidium alipioi* chromosomes (Figure 3e). Conversely, the CgB probe showed hybridization signals on the W chromosome of *Characidium alipioi* (Figure 3d).

### Distribution of repetitive DNA probes

**Figure 4.**
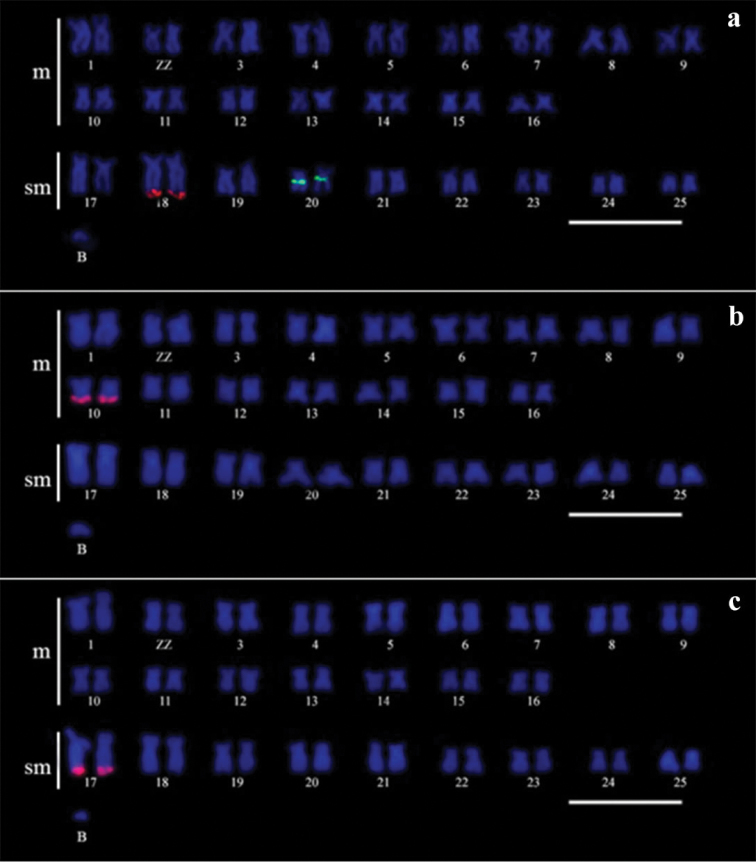
Karyotypes of *Characidium alipioi* arranged from mitotic metaphases after FISH with repetitive DNA probes. **a** 18S and 5S rDNA probes **b** histone H3 probe, and **c** U2 snRNA probe. Bar =10 µm.

FISH experiments on the *Characidium alipioi* chromosome preparations using a 18S rDNA probe revealed sites of this gene in the terminal position of autosomal pair No. 18, whereas the 5S rDNA was mapped in the pericentromeric region of chromosome pair No. 20 (Figure 4a). Histone H3 sites were found in the m chromosome pair No. 10 (Figure 4b), whereas the U2 snRNA gene was located on the sm chromosomes pair No. 17 (Figure 4c).

**Figure 5.**
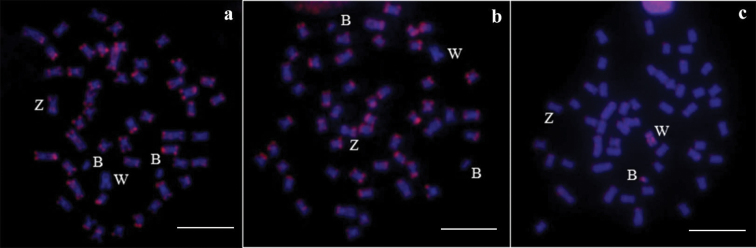
Mitotic metaphases after FISH with the microsatellites. **a** (GA)_15_ **b** (CA)_15_ and **c** (GAG)_10_. Note the accumulation of (GAG) 10 in the B and W chromosomes.

FISH with probes containing the microsatellite sequences (CA)_15_ and (GA)_15_ in the *Characidium alipioi* chromosomal preparations showed similar patterns of distribution with preferential accumulation in the terminal regions of the chromosomes, except for the W and B chromosomes (Figure 5a and b), which had lower abundance and weak hybridization signals. Furthermore, the intense signals of the microsatellite (GAG)_10_ showed preferential accumulation on the W and B chromosomes of this species (Figure 5c).

## Discussion

The occurrence of B chromosomes in *Characidium alipioi* genome was revealed for the first time in this study, particularly since the population of *Characidium alipioi* of Santa Bárbara do Tugúrio-MG analyzed by Pansonato-Alves et al. (2014) showed no extra chromosomes. The presence of these elements in only certain populations was observed in other species of the genus *Characidium* such as *Characidium zebra*, *Characidium pterostictum* and *Characidium gomesi* (Pansonato-Alves et al. 2014). Our data does not allow to conclude about the dynamics or relationship of these chromosomes between populations; however, at least two mechanisms, which are based on the findings of two model organisms have been proposed to be involved in the absence of B chromosomes in certain populations: geographical barriers that limit the spread of individuals with these elements in *Eyprepocnemis plorans* Charpentier, 1825 (Cabrero et al. 1997, Manrique-Poyato et al. 2015) and the close relationship between temperature and rainfall factors that could influence the variations in the presence of B chromosomes, as observed in *Myrmeleotettix maculatus* Thunberg, 1815 (Hewitt and John 1967; Hewitt and Ruscoe 1971).

The individuals of the same species and location included in this study were analyzed in a previous study by Centofante et al. (2003); however, these authors did not detect B chromosomes in their analysis. Although cytogenetic studies in *Characidium* have revealed the occurrence of supernumerary chromosomes restricted to certain populations of some species (Pansonato-Alves et al. 2014), it should be noted that the geographical proximity of the sampling sites and the time between the present analysis and the analysis by Centofante et al. (2003) suggest that the two samples do not belong to different populations. Similarly, it can’t be stated that the B chromosomes have arisen in this population after the first study. However, because some individuals with no B chromosomes were also identified in the present study, it is likely that the samples analyzed in the 2003 study were formed by individuals who do not carry these extra elements.

Chromosome painting using the B chromosomes of *Characidium alipioi* (CAB) as the probe indicated shared sequences between the A and B complements and, more specifically, the pericentromeric region of a sm chromosome pair (pair No. 19). This result probably reflects a relationship between these chromosomes and intraspecific origin of the supernumerary chromosomes in genome of this species and that pair No. 19 is likely the chromosome of its origin. Previous studies have identified the origin of B chromosomes from the A complement of the host species (Bugrov et al. 2003; De Jesus et al. 2003; Lamb et al. 2005; Teruel et al. 2010; Peng et al. 2011; Silva et al. 2014; Valente et al. 2014; Utsunomia et al. 2016); however, in a few cases it was possible to identify the ancestral chromosome, such as in the grasshopper (Teruel et al. 2010; Bueno et al. 2013) and in two fish species *Astyanax paranae* Eigenmann, 1914 (Silva et al. 2014) and *Moenkhausia sanctaefilomenae* Steindachner, 1907 (Utsunomia et al. 2016).

The intraspecific origin of B chromosomes has been reported in other species of the genus *Characidium*, namely *Characidium pterostictum*, *Characidium gomesi* and *Characidium* sp. aff. *Characidium vidalli* (Pansonato-Alves et al. 2014; Schacchetti et al. 2015). However, because in these species this chromosome originated from the Z and W chromosomes and the CaB probe in the present study showed no signals on these chromosomes, these supernumerary elements do not seem to share the same ancestral supernumerary chromosomes that are present in *Characidium alipioi*. In addition, the *Characidium alipioi* B chromosomes showed no homology with the supernumeraries of *Characidium gomesi* and *Characidium oiticicai*, which are two species whose B chromosomes are apparently composed of different types of repetitive DNA and have different origins (Pansonato-Alves et al. 2014). Therefore, it possible that the supernumerary elements in *Characidium alipioi* originated independently from the other types of B chromosomes reported in the representatives of this genus, and the phylogenetic position of these species at different clades (Figure 1) supports this assumption. However, these conclusions should be made with caution because the probes obtained by microdissection are composed of anonymous sequences, and the amplification method (GenomePlex) may favor specific sequences of repetitive DNA that are present in the B chromosomes and absent on other A chromosomes. In this context, it cannot be ruled out that other sequences shared between A and B chromosomes are not represented in the probes used in this study, which was previously noted by Pansonato-Alves et al. (2014).

Hybridizations with microsatellite DNA sequences demonstrated the presence of these repetitive elements in B chromosomes. (GA)_15_ and (CA)_15_ are both dispersed with conspicuous blocks in the terminal regions of the A chromosomes and are less abundant in the supernumerary chromosomes. Moreover, a clear accumulation was observed with respect to the (GAG)_10_ sequence. Notably, the hybridization with the (GAG)_10_ probe revealed a preferential accumulation in B and W chromosomes in *Characidium alipioi*. Similarly, microsatellites were detected in the B chromosomes of maize (Ananiev et al. 2005), rye (Langdon et al. 2000) and locusts (Milani and Cabral-de-Mello 2014; Ruiz-Ruano et al. 2015). Given that the CaB probe did not paint the *Characidium alipioi* sex chromosomes, a possible explanation for the accumulation of (GAG)_10_ in the B and W chromosomes would be that the CaB probe does not contain this microsatellite, which probably occurred due to the amplification method used in this study, as mentioned above. However, if it is considered that the accumulation mechanisms of this type of repetitive DNA permit its fixation on certain chromosomes due to its non-recombinant nature and preferential accumulation in heterochromatic regions, as observed in other studies (Cuadrado and Jouve 2011, Lohe et al. 1993, Scacchetti et al. 2015), then the distribution of microsatellites in these chromosomes may not reflect the aspects of its origin, but its accumulation after the appearance of the B chromosomes.

Our present results extend the knowledge of the structure and composition of B chromosomes between representatives of the *Characidium* genus, particularly in *Characidium alipioi*. In addition, the shared sequences between the A and B chromosomes of this species suggests an intraspecific origin of these chromosomes that is independent from the B chromosomes of other congeneric species. These observations reinforce the idea that this fish group is an interesting model to study the origin and structure of B chromosomes.

## Supplementary materials

Supplementary material 1 Table S1 Data type: molecular data Explanation note: Average distance between the CytB sequences of the *Characidium* species. This dataset is made available under the Open Database License (http://opendatacommons.org/licenses/odbl/1.0/). The Open Database License (ODbL) is a license agreement intended to allow users to freely share, modify, and use this Dataset while maintaining this same freedom for others, provided that the original source and author(s) are credited.Érica Alves Serrano, Ricardo Utsunomia, Patrícia Sobrinho Scudeller, Claudio Oliveira, Fausto Foresti

Supplementary material 2 Table S2 Data type: molecular data Explanation note: Average distance between the COI sequences of the *Characidium* species. This dataset is made available under the Open Database License (http://opendatacommons.org/licenses/odbl/1.0/). The Open Database License (ODbL) is a license agreement intended to allow users to freely share, modify, and use this Dataset while maintaining this same freedom for others, provided that the original source and author(s) are credited.Érica Alves Serrano, Ricardo Utsunomia, Patrícia Sobrinho Scudeller, Claudio Oliveira, Fausto Foresti
